# Effects of β-glucosidase and α-rhamnosidase on the Contents of Flavonoids, Ginkgolides, and Aroma Components in Ginkgo Tea Drink

**DOI:** 10.3390/molecules24102009

**Published:** 2019-05-25

**Authors:** Xianying Fang, Yurong Dong, Yingying Xie, Lei Wang, Jingqiu Wang, Yuechen Liu, Linguo Zhao, Fuliang Cao

**Affiliations:** 1 College of Chemical Engineering, Nanjing Forestry University, Nanjing 210037, China; fxy_08@163.com (X.F.); 18260077753@163.com (Y.D.); dawntalexyy@163.com (Y.X.); wl0308njfu@163.com (L.W.); 18761687786@163.com (J.W.); 2 School of Science, Rensselaer Polytechnic Institute, New York, NY 12180, USA; liuy42@rpi.edu; 3 Co-Innovation Center for Sustainable Forestry in Southern China, Nanjing Forestry University, Nanjing 210037, China; cao@njfu.edu.cn

**Keywords:** ginkgo tea, glycosidase, flavonoid, ginkgolide, aroma component, anti-oxidation, anti-inflammation, anti-tumor

## Abstract

Ginkgo tea is a kind of health food produced from *Ginkgo biloba* leaves. The market of Ginkgo tea encountered many difficulties because of its bad palatability and vague function statement. In this study, two kinds of glycosidase were used to improve the flavor of Ginkgo tea, and three kinds of bioactivities were selected to investigate the health care function of the tea infusion. The aroma components extracted by headspace absorb (HSA) method during the making of Ginkgo tea were analyzed by GC-MS. The flavonoids and ginkgolides released into the tea infusion were studied by HPLC. A combination of β-glucosidase (β-G) and α-rhamnosidase (α-R) was applied during the making of the tea. The contents of characteristic aroma components and the release of total flavonoids and ginkgolides were increased significantly by adding β-G and α-R. The composition of flavone glycosides was changed greatly. The free radical scavenging, inhibition of inflammatory cell activation, and tumor cytotoxicity activities of the tea were demonstrably improved. According to the release of active components, Ginkgo tea can be brewed repeatedly for at least three times. The enzymes used here show potential application prospects in the making of Ginkgo tea or tea drink to get higher contents of flavonoids, ginkgolides, and aroma components.

## 1. Introduction

*Ginkgo biloba* is popular plant with long history, and is widespread in China, Europe, America, and other countries. Ginkgo products are welcomed by people from all over the world, such as EGB761 in the early days [1]. Tea has been consumed for thousands of years as a daily health drink in China and has been traditionally used as a medication based on experience. China and Japan have also described its biological activities including extensive antioxidant and antitumor activities of the active ingredients [2,3]. Ginkgo tea, which is made from ginkgo leaves, is healthy. The main active ingredients of ginkgo tea are ginkgo flavonoid, ginkgolides, and some other glycosides [4]. Although ginkgo tea is thought to be good for health [5], the flavor components and active compounds released during the making of the tea are not systematically studied [6]. The typical aroma components emitted from ginkgo leaf include benzyl alcohol, phenethyl alcohol, β-Ionone, Limonene, Vanillic acid, and so on [7,8]. Ginkgo flavonoids usually exist in the glycoside form in nature, including the aglycones of quercetin, isorhamnetin, kaempferol, and so on [9]. Many pharmaceutical companies have approved ginkgolides’ contribution to protecting the nervous system and cardiovascular system [10]. However, it is not clear whether ginkgolide can be released during the making of ginkgo tea.

The compact cellulose structure is one of the natural barriers for the release of aroma components, flavonoids, and ginkgolides from ginkgo tea [11]. Several researches showed that bio-enzymatic treatment can promote the release of bioactive compounds from the complex plant fiber structure [12,13,14]. Although it is difficult to draw the general impact of glycosylation on ginkgo flavonoids’ biological benefits, O-glycosylation may reduce the bioactivity (such as anti-oxidation, anti-inflammation, and anti-tumor) of these compounds [15,16,17,18]. The main glyco-ligands in ginkgo flavonoids are composed of glucose or rhamnose, or both [19,20]. β-glucosidase and α-rhamnosidase are two common glycoside hydrolases, which are reported to be able to finish the enzymatic de-glycosylation of many nature flavone glycosides [16,21,22].

When making tea, the water temperature is usually up to 90 °C. However, common enzymes lose activity at such a high temperature [23]. In our previous study, one kind of thermo-stable β-glucosidase from *Thermotoga petrophila* DSM13995 and another kind of α-rhamnosidase from *Aspergillus terreus* CCF 3059 were prepared to hydrolyze special glycosyl groups under high temperature [24,25]. Thus, the treatment by thermo-stable bio-enzymes might improve the flavor and bioactivity of ginkgo tea. European Regulation 1924/2006 states that all health claims made on foods need to be substantiated scientifically [26]. Here, the bioactive compounds in tea infusion and biological activity of ginkgo tea, especially with the treatment of glycosidase, were systematically studied.

## 2. Results and Discussion

### 2.1. Ginkgo Tea Can Be Brewed Repeatedly for at Least Three Times Considering the Release of Flavonoids and Ginkgolides

**Figure 1:**
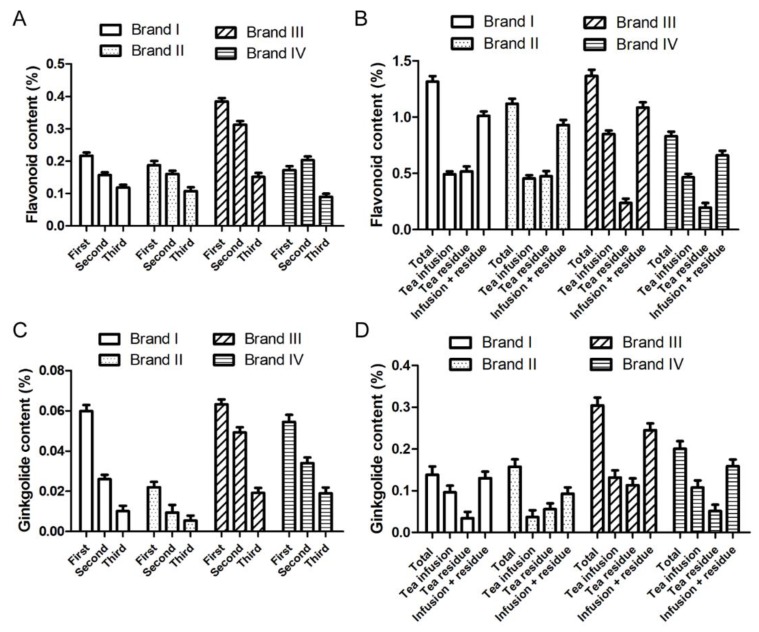
The amount of flavonoids and ginkgolides released into the Ginkgo tea infusion. Four kinds of Ginkgo tea were purchased from the market and each Ginkgo tea was brewed for three times. (**A**) The total flavonoid content in the tea infusion of each time was determined by HPLC-UV analysis. (**B**) The total flavonoid content in the Ginkgo tea, the tea infusion, and the tea residue after three times brewing were determined by HPLC-UV analysis. (**C**) The ginkgolide content in the tea infusion of each time was determined by HPLC-ELSD analysis. (**D**) The ginkgolide content in the Ginkgo tea, the tea infusion, and the tea residue after three times of brewing were determined by HPLC-ELSD analysis. Data are mean ± SD of three independent experiments.

Usually, when making Chinese tea, the tea will be brewed for at least two or three times. Some unwanted substances will release from the tea and the taste becomes bad after brewing too many times. According to the product instruction of Ginkgo tea, it can be brewed at least three times. Considering the healthcare function of Ginkgo tea, the focus should be active compounds releasing into the water. Four kinds of ginkgo tea were selected and studied for the amount of total flavonoids and ginkgolides releasing into the water during the making of the tea. The results indicated that flavonoids were released into the tea infusion every time the tea was brewed with the same material (Figure 1A), and that the release amount of the last time accounted for 18–24% of the total release. For two kinds of powder-tea (brand III and IV), over 60% flavonoids were released into the tea infusion by brewing three times totally, while only less than 50% flavonoids were released into the water of leaf-tea (brand I and II) (Figure 1B). Although ginkgolides are thought to be difficult to dissolve in water at room temperature, all the four kinds of ginkgolides (ginkgolide A, B, C, and bilobalide) were released gradually during tea-making (Figure 1C, Figure S1). Most ginkgolides were released during the first two times of brewing, and the release amount of the last time accounted for 10–18% of the total release. The total release was up to 70% (brand I) of the total ginkgolides in ginkgo tea (Figure 1D). Considering the moderate dissolution rate of flavonoids and ginkgolides, Ginkgo tea of brand I was selected for further study.

### 2.2. The Addition of Glycosidase When Making the Tea Promotes the Release of Flavonoids and Aroma Components from the Ginkgo Tea

As mentioned before [27], the number and kind of glycoside in the side chain may influence the bioactivity of flavonoids. Since most flavonoids exist in glycoside form in ginkgo leaves, proper treatment by glycosidase may improve the function of ginkgo products. Quercetin-3-*O*-rutinoside (rutin) and kaempferol-3-*O*-rutinoside glycosides are two free flavone glycosides with relatively high content in ginkgo leaves [28]. The hydrolysis effect of glycosidase on these two glycosides provided reference for the enzymatic treatment of Ginkgo tea. In our previous research, kaempferol-3-*O*-rutinoside could be completely converted to kaempferol using TpeBgl3 and AteRha78 [16]. The conversion rate of rutin to quercetin using different combinations of three β-glucosidases (β-G) and α-rhamnosidases (α-R) in our laboratory were compared. The results indicated that the combination of TpeBgl3 and AteRha78 had the highest conversion rate (95%) under the same enzyme dosage (Table S1).

**Figure 2:**
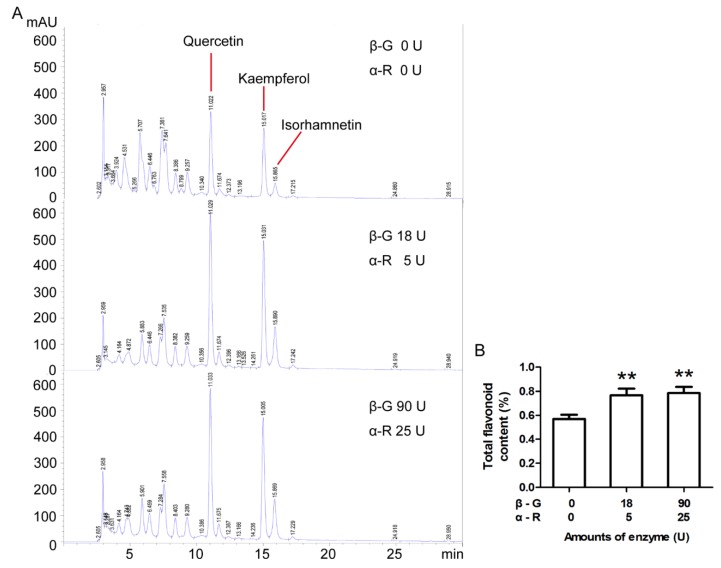
The addition of β-G and α-R during the making of the tea increased the release of ginkgo flavonoids. Ginkgo tea (4 g) was put into a heat preservation cup, 200 mL boiling water was added into the same cup and kept warm for 15 min. α-R was added and kept at 65 °C for 1 h, then β-G was added and kept at 90 °C for 1 h. Tea infusion of the first time was collected by filtration. An amount of 200 mL boiling water was added into the residue of Ginkgo tea and kept warm for 15 min for the second time. Tea infusion of the second time was collected by filtration. The above step was repeated for the third time, and the tea infusion of the third time was collected by filtration. Before HPLC detection, the tea infusion was concentrated and dried, then dissolved in methanol. (**A**) The contents of quercetin, kaempferol, and isorhamnetin were determined by HPLC analysis, and (**B**) the content of total ginkgo flavonoids was calculated according to these three flavonoid aglycones. Data are mean ± SD of three independent experiments. ** *p* < 0.01 vs. control.

**Figure 3:**
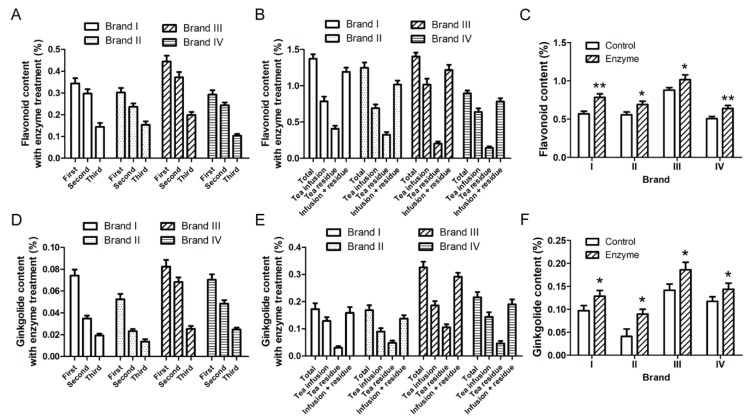
The addition of β-G (0.125 U/mL) and α-R (0.45 U/mL) during the making of the tea increased the release of ginkgo flavonoids and ginkgolides. Ginkgo tea (4 g) was put into a heat preservation cup, 200 mL boiling water was added into the same cup and kept warm for 15 min. α-R was added and kept at 65 °C for 1 h, then β-G was added and kept at 90 °C for 1 h. Tea infusion of the first time was collected by filtration. An amount of 200 mL boiling water was added into the residue of Ginkgo tea and kept warm for 15 min for the second time. Tea infusion of the second time was collected by filtration. The above step was repeated for the third time, and the tea infusion of the third time was collected by filtration. The experiments in the control group were taken with the same steps except without enzyme addition. Before HPLC detection, the tea infusion was concentrated and dried, then dissolved in methanol. (**A**) The total flavonoid content in the tea infusion of each time was determined by HPLC-UV analysis. (**B**) The total flavonoid content in the Ginkgo tea, the tea infusion, and the tea residue after three times of brewing were determined by HPLC-UV analysis. (**C**) The total flavonoids released from the Ginkgo tea during three times of brewing were compared between the control and enzyme treatment groups. (**D**) The ginkgolide content in the tea infusion of each time was determined by HPLC-ELSD analysis. (**E**) The ginkgolide content in the Ginkgo tea, the tea infusion, and the tea residue after three times of brewing were determined by HPLC-ELSD analysis. (**F**) The total ginkgolides released from the Ginkgo tea during three times of brewing were compared between control and enzyme treatment group. Data are mean ± SD of three independent experiments. Data are mean ± SD of three independent experiments. * *p* < 0.05, ** *p* < 0.01 vs. control.

To determine the best enzyme dosage for the treatment of Ginkgo tea, the conversion rates of rutin to quercetin at different concentrations of β-G and α-R were compared. According to our calculation, the content of rutin in Ginkgo tea is about 0.1%. Take 10 mg rutin as the substrate, over 80% rutin can be converted to quercetin in 1 h by 0.125 U/mL (25 U in total) β-G and 0.45 U/mL (90 U in total) α-R (Figure S2). Here, the theoretical 100% yield of quercetin was calculated according to the molar number of rutin. According to the enzyme concentration used in rutin hydrolysis, two glycosidases were added into the water in turn during the making of the tea, and the Ginkgo tea was brewed three times as mentioned in Figure 1. Contents of total flavonoids released into the tea infusion were calculated. The results indicated that the addition of β-G (0.125 U/mL) and α-R (0.45 U/mL) during the making of the tea could increase the total flavonoids content in the tea infusion significantly (Figure 2). The tea infusion with enzyme (0.125 U/mL β-G and 0.45 U/mL α-R) treatment was applied for HPLC analysis of ginkgo flavonoids and ginkgolides. The data in Figure 3 and Figure S3 show that the amounts of flavonoids and ginkgolides released into the tea infusion of four brands were all increased after enzyme treatment compared to the control group.

In addition, the release amount of the characteristic aroma components during the making of the tea was also analyzed. Ginkgo tea (4 g) was put into a heat preservation cup, 200 mL boiling water was added into the same cup and kept warm for 15 min. α-R (0.45 U/mL) was added and kept at 65 °C for 1 h, then β-G (0.125 U/mL) was added and kept at 90 °C for 1 h. The tea infusion was filtered immediately into separating funnel and kept closed. After the tea infusion was cooled, aroma components in the tea infusion were extracted using ether. The results indicated that the addition of enzyme during the making of the tea increased the total amount of aroma components in the tea infusion (Table 1).

A possible reason for the increase is that when the glycosidic bonds in the plant fiber structure are cut off by β-G and α-R, the gap between the natural barriers becomes larger. As a result, it is easier for the flavonoids, ginkgolides, and aroma components to release. Additionally, some flavonoids, ginkgolides, and aroma components may be linked to the side chain of certain structure via glycosidic bonds. When the glycosidic bonds are hydrolyzed by β-G and α-R, these components are released.

### 2.3. The Addition of Glycosidase during the Making of the Tea Increases the Content of Flavone Aglycone in the Tea Infusion

Although the addition of 0.45 U/mL β-G and 0.125 U/mL α-R can increase the release of total flavonoids and aroma components from the Ginkgo tea, it is not enough for the conversion of flavone glycoside in the tea infusion (date are not shown). According to the total flavonoids content in Ginkgo tea (0.8–1.5%), three groups of enzyme concentration were compared for the transformation effect on the flavonoids in Ginkgo tea infusion. When 7.5 U/mL β-G and 2 U/mL α-R were added during the making of the ginkgo tea, rutin was thoroughly hydrolyzed, kaempferol-3-O-rutinoside could not be converted, and quercetin, kaempferol, and isorhamnetin almost reached the highest yields (Figure S4). So, 7.5 U/mL β-G and 2 U/mL α-R were used for the treatment of Ginkgo tea.

**Figure 4:**
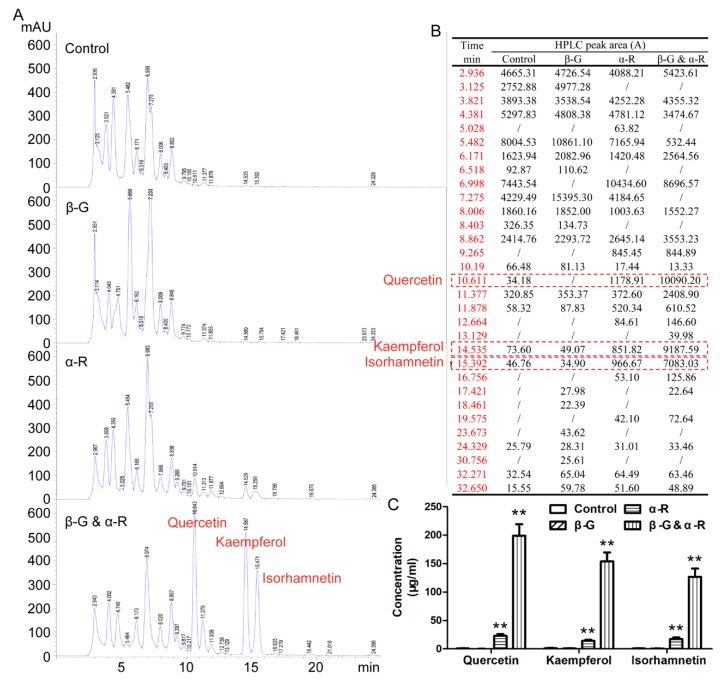
Effect of different enzyme combination on the composition of characteristic flavonoids in the tea infusion. Ginkgo tea (4 g) was put into a heat preservation cup, 200 mL boiling water was added into the same cup and kept warm for 15 min. α-R (2 U/mL) was added and kept at 65 °C for 1 h, then β-G (7.5 U/mL) was added and kept at 90 °C for 1 h. Tea infusion was collected by filtration. The experiments in the control group were taken with the same steps except without enzyme addition. The experiments in single enzyme groups were taken with the same steps except that only one kind of enzyme was added. Before HPLC detection, the tea infusion was concentrated and dried, then dissolved in methanol. (**A**) Composition analysis of flavonoids in the tea infusion by HPLC. (**B**) The peak area of flavonoids in the tea infusion. (**C**) The content of quercetin, kaempferol, and isorhamnetin in the tea infusion. ** *p* < 0.01 vs. control.

To compare the influence of β-G and α-R on the flavonoids in the tea infusion, β-G, α-R, or both were added, respectively, to the system during the making of the tea. Under the setting condition for HPLC detection, the retention time of flavone glycoside was usually shorter than its flavone aglycone. If the flavone glycosides were same in the structure except for the end glycosyl group and had only one sugar base difference, the retention time were likely very close. The addition of α-R or both β-G and α-R can increase the release of three major ginkgo flavone aglycones (Quercetin, Kaempferol, and Isorhamnetin) (Figure 4). Although no obvious changes were found with the HPLC chromatogram after the treatment of β-G, it does mean there were no changes with the flavone glycoside components in the tea infusion. Because the increase of ginkgo aglycone by the treatment of both β-G and α-R are several times more than that of only α-R, it can be deduced that in addition to the increase in aglycone, there must be some changes in the flavone glycosides that were not reflected in the HPLC chromatogram.

To further confirm the influence of β-G and α-R on the composition of flavonoids in Ginkgo tea, the tea infusion making from all the four brands were analyzed by HPLC. Our results indicated that the flavone aglycones (quercetin, kaempferol, and isorhamnetin) in the tea infusion were increased by the treatment of β-G and α-R in all the four kinds of Ginkgo tea and in each time of brewing (Table 2). There were significant differences in the dissolution of flavonoid aglycones. In the leaf-tea (brand I and II), flavonoids aglycones were released into the water in batches by three times of brewing, while most flavonoid aglycones were released into the water during the first time of brewing in the powder-tea (brand III and IV). It should be noticed that the content of quercetin, kaempferol, and isorhamnetin in the tea infusion with enzyme treatment here did not represent the total content of flavone aglycones in Ginkgo tea. Many flavone aglycones still existed in their glycoside forms, which were not free. Based on the characteristic of the enzyme used here, only those ending with glucose or rhamnose were able to be de-glycosylated.

### 2.4. The Addition of Glycosidase during the Making of the Tea Promotes the Health Care Function of Ginkgo Tea

To our knowledge, the bioactivity of Ginkgo tea has never been systematically studied, especially regarding the tea infusion. Ingredients released when making the tea are different from EGB (Extract of *Ginkgo biloba*), so the healthcare function of Ginkgo tea is worth being carefully studied. In the market, Ginkgo tea is welcomed for its possible health care function, especially on the prevention and treatment of disease in the cardio-cerebrovascular and nervous system [29,30]. Numerous studies indicate that cardio-cerebrovascular and nervous system diseases are closely related to the oxidation and inflammation in vivo [31,32]. From the data in Figure 1, we know that both flavonoids and ginkgolides were released into the tea infusion. Ginkgolides, especially ginkgolide B, are well known for protecting the body from cardio-cerebrovascular disease, anti-inflammation, anti-oxidation, and some other pharmacological activities, and they have been approved for clinical application [33,34]. Ginkgo flavonoids show more extensive physiological activities, such as anti-oxidation, anti-inflammation, anti-tumor, anti-aging, and so on [35,36].

**Figure 5:**
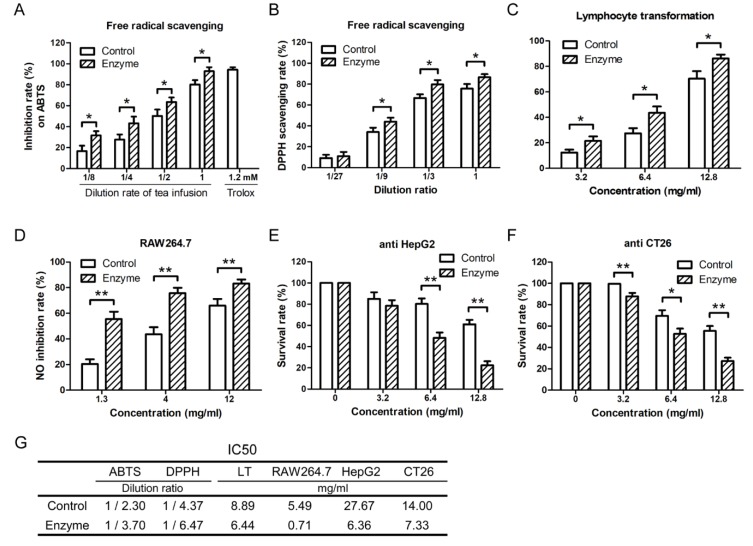
The free radical scavenging, inhibition of inflammatory cell activation, and tumor cytotoxicity activities of Ginkgo tea were improved by the addition of bio-enzymes during the making of the tea. Ginkgo tea (4 g) was put into a heat preservation cup, 200 mL boiling water was added into the same cup and kept warm for 15 min. α-R (2 U/mL) was added and kept at 65 °C for 1 h, then β-G (7.5 U/mL) was added and kept at 90 °C for 1 h. The Ginkgo tea was brewed three times as mentioned before. The experiments in the control group were taken with the same steps except without enzyme addition. Tea infusion was collected by filtration, then concentrated and dried, and dissolved in DMSO. The Ginkgo tea infusion treated with or without enzyme was used for the detection of (**A**) total antioxidant capacity against the oxidation of ABTS and (**B**) DPPH scavenging ability. (**C**) The concentrate of Ginkgo tea infusion was used for the assay of inhibition ability against Con A-induced lymphocyte proliferation and (**D**) LPS-induced NO release in RAW264.7 cells. (**E**,**F**) The concentrate of Ginkgo tea infusion was used for the assay of inhibition ability against the proliferation of HepG2 and CT26 cells. (**G**) The IC_50_ value of free radical scavenging, inhibition of inflammatory cell activation, and tumor cytotoxicity activities. The concentration unit ‘mg/mL’ means that x mg dry raw material (Ginkgo tea) per milliliter. Data are mean ± SD of three independent experiments. * *p* < 0.05, ** *p* < 0.01.

Theoretically speaking, glycosidase will not change the ginkgolides components in the tea infusion. However, the composition of flavonoids in the tea infusion was changed greatly by glycosidase. So, the anti-oxidation, anti-inflammation, and anti-tumor activities of tea with or without adding glycosidase were studied. Before the bioactivity of Ginkgo tea was studied, safety research was applied on mouse lymphocytes. The concentrates of Ginkgo tea infusion did not show cytotoxicity on the normal lymphocytes regardless of treating with enzyme or not (Figure S5). Our data indicated that the tea infusion showed higher free radical scavenging activity against DPPH and ABTS after β-G and α-R treatment (Figure 5A,B). The inhibition rate against LPS-induced NO release of RAW264.7 and Con A-activated lymphocytes proliferation were increased by enzyme addition (Figure 5C,D). The in vitro proliferation of HepG2 and CT26 tumor cells were also inhibited stronger by the tea infusion concentrates treated with enzyme (Figure 5E,F). Totally speaking, the addition of β-G and α-R during the making of Ginkgo tea showed the potential to improve the anti-oxidation, anti-inflammation, and anti-tumor activities (Figure 5G).

## 3. Materials and Methods

### 3.1. Materials

Four brands of Ginkgo tea (Hubei (I), Jiangsu (II), Zhejiang (III), and Shandong (IV)) were purchased from Alibaba (Hangzhou, China). Standard substances (flavonoids and ginkgolides), DPPH, LPS, Concanavalin A (Con A), and MTT were purchased from Sigma-Aldrich (St. Louis, MO). Total antioxidant capacity test kit (ABTS method) was purchased from Beyotime (Shanghai, China). DMEM, 1640 medium, and fetal bovine serum were purchased from Gibco (Grand Island, NY). Immobilized metal affinity column was purchased from Novagen (Madison, WI). DEAE SFF column was purchased from GE healthcare (Buckinghamshire, UK). All β-glucosidase and α-rhamnosidase were screened and prepared in our own laboratory. Methanol was HPLC grade, and all other solvents and chemicals were analytical grade.

### 3.2. Animals

Six-week-old female Balb/c mice were purchased from Comparative Medical Center of Yangzhou University (Yangzhou, China). They were maintained with free access to pellet food and water in plastic cages at 21 ± 2 °C and kept on a 12 h light/dark cycle. The procedures were approved by the local ethics committee of Nanjing Forestry University and in accordance with the Helsinki Declaration of 1975, as revised in 2008. All efforts were made to minimize the animals’ suffering and to reduce the number of animals used.

### 3.3. Cell Culture

Murine lymphocytes and RAW264.7 cells were maintained in RPMI 1640 medium. Human hepatoma HepG2 cells and murine colon CT26 cells were maintained in DMEM medium. All media were supplemented with 10% fetal bovine serum plus 2 mM glutamine, 100 U/mL penicillin, and 100 mg/mL streptomycin. Lymphocytes were obtained from the lymph node of Balb/c mice. Other cell lines were purchased from Shanghai Institute of Cell Biology (Shanghai, China), and were cultured at 37 °C in 5% (*v*/*v*) CO_2_ atmosphere.

### 3.4. Enzyme Preparation

Recombinant plasmids of β-glucosidase pET20b-TpeBgl3 (GH3 *Thermotoga petrophila* DSM13995), pET20b-TthBgl3 (GH3 *Thermotoga thermarum* DSM 5069), pPICZαA-AniBgl3 (GH3 *Aspergillus niger* NL-1), and recombinant plasmids of α-rhamnosidase pPICZαA-AteRha78 (GH78 *Aspergillus terreus* CCF 3059), pET20b-AniRha78 (GH78 *Aspergillus niger* NL-1), and pET28a-BthRha (*Bacteroides thetaiotaomicron* VPI 5482) were all constructed in our laboratory. The information on the enzyme type, characteristics, activity, substrate specificity, temperature dependence, heat stability, and the experimental data are presented in Table 3. The preparation of the above glycosidase was done as described before [23,24,25,37,38,39].

Briefly, recombinant bacteria with prokaryotic expression vectors (pET vector) was activated in LB plates (*Kan* or *Amp* resistance) at 37 °C for 1 d, single colony was inoculated into 4 mL LB liquid medium and incubated at 37 °C, 180 r/min for 8 h, bacteria liquid was transferred into 150 mL LB liquid medium and incubated at 30 °C, 180 r/min for 10 h, bacteria cells were harvested by centrifuge. Recombinant bacteria with eukaryotic expression vectors (pPICZαA vector) was inoculated into YPD plate and incubated at 28 °C for 2–3 d, single colony was inoculated into 30 mL BMGY medium and incubated at 28 °C for 24–48 h, cells were inoculated into 50 mL BMMY medium according to the initial inoculum size of OD600 = 1.0 and incubated at 28 °C, 180 r/min, 0.6% methanol was added to induce the production of enzyme every 24 h; after 15 days of incubation, the supernatant was harvested as crude enzyme.

**Figure 6:**
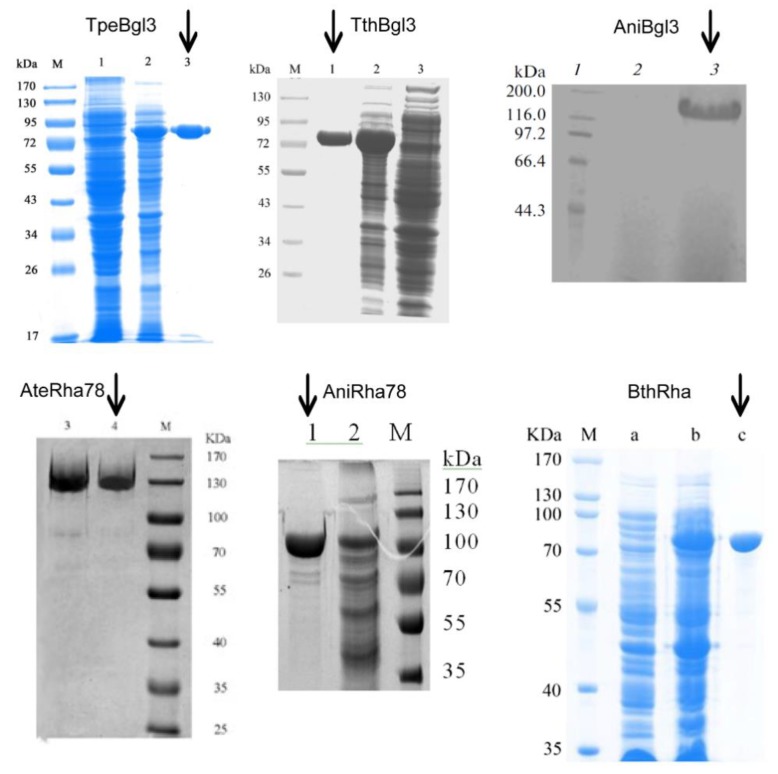
SDS-PAGE analysis of the purity of final product. Lane M, protein molecular mass marker. TpeBgl3: lane 1, the crude extract of *E. coli* BL21 (DE3) harboring pET-20b; lane 2, the crude extracts of *E. coli* BL21 (DE3) harboring pET-TpeBgl3; lane 3, TpeBgl3 purified by Ni–NTA resin affinity chromatography. TthBgl3: lane 1, purified TthBgl3; lane 2, cell-free extract of JM109 (DE3) harboring pET-20-TthBgl3; lane 3, cell-free extract of JM109 (DE3) harboring pET-20b. AniBgl3: lane 2, supernatant of *P. pastoris*/pPICZαA as negative control; lane 3, the purified recombinant β-glucosidase. AteRha78: Lane 3, crude enzyme; lane 4, purified enzyme. AniRha78: lane 1, pure enzyme; lane2: crude enzyme. BthRha: land a, cell-free extract of BL21(DE3) harboring pET28a; land b, cell-free extract of BL21(DE3) harboring pET28a-BthRha, land c, BthRha purified by Ni-NTA affinity.

Purification: TpeBgl3 and TthBgl3 were purified by heat treatment and Ni-NTA affinity chromatography; AniRha78 and BthRha were purified by Ni-NTA affinity chromatography; AteRha78 induced expression of crude enzyme solution by ammonium sulfate precipitation, anion exchange resin, and dialysis, obtaining an electrophoretically pure protein of interest; AniBgl3 was secreted into the supernatant and was harvested from 120 h culture of recombinant *P. pastoris* by centrifugation, then the recombinant β-glucosidase was purified by a simple precipitation. The purity of each enzyme is shown in Figure 6.

### 3.5. Making Ginkgo Tea

Ginkgo tea (4 g) was put into a heat preservation cup, 200 mL boiling water was added into the same cup and kept warm for 15 min for the first time. Tea infusion of the first time was collected by filtration. An amount of 200 mL boiling water was added into the residue of Ginkgo tea and kept warm for 15 min for the second time. Tea infusion of the second time was collected by filtration. The above step was repeated for the third time, and the tea infusion of the third time was collected by filtration. Before HPLC detection, the tea infusion was concentrated by rotary evaporator and dried by vacuum freeze dryer, then dissolved in methanol.

### 3.6. Extraction of Total Ginkgo Flavonoids in Ginkgo Tea and the Residue of Ginkgo Tea

According to the Chinese Pharmacopoeia, 2.5 g Ginkgo tea was firstly degreased with chloroform by hot reflux extraction, the chloroform extract was removed by filtration. The dry Ginkgo tea residue was extracted by hot reflux of methanol. The methanol extract was dried by vacuum concentration and then treated by hot reflux of 40 mL CH_3_OH–25%HCl (4:1) solution. Before HPLC analysis, the acid hydrolysate of Ginkgo tea extract was diluted by methanol to a suitable detection concentration.

### 3.7. Analysis of Ginkgo Flavonoids

Flavonoids (Quercetin, Kaempferol, and Isorhamnetin) were analyzed by Agilent 1260 Series HPLC, using Eclipse XDB-C18 chromatographic column, UV detector, gradient elution by mobile phase of methanol–water system (0~30 min: 50%CH_3_OH + 50%H_2_O ⟶ 80%CH_3_OH + 20%H_2_O, 30~33 min: 80%CH_3_OH + 20%H_2_O ⟶ 50%CH_3_OH + 50%H_2_O, 0.8 mL/min), detection wavelength of 360 nm. The contents of quercetin, kaempferol, and isorhamnetin were calculated according to authentic compounds. The content of total flavonoid was calculated by the following formula [40]:

Total flavonoids (%) = (Quercetin + Kaempferol + Isorhamnetin)% × 2.51 (1)

### 3.8. Extraction of Ginkgolides in Ginkgo Tea and the Residue of Ginkgo Tea

According to the Chinese Pharmacopoeia, 2.5 g Ginkgo tea were degreased with ligarine by hot reflux extraction, the ligarine extract was removed by filtration. The dry Ginkgo tea residue was extracted by hot reflux of methanol. The methanol extract was dried by vacuum concentration. Before HPLC anlysis, the Ginkgo tea extract was diluted by methanol to a suitable detection concentration.

### 3.9. Analysis of Ginkgolides

Ginkgolides (ginkgolide A, B, C, and bilobalide) were analyzed by Agilent 1260 Series HPLC, using Eclipse XDB-C18 chromatographic column, ELSD detector, constant proportional elution by mobile phase of methanol–water system (0~30 min: 33%CH_3_OH + 67%H_2_O, 0.8 mL/min), CSChrom Plus 3.6 Chromatographic workstation, the drift tube temperature 100 °C, and the carrier gas velocity 2.2 L/min. The content of total flavonoid was calculated by the following formula:

Total ginkgolides (%) = (Ginkgolide A + Ginkgolide B + Ginkgolide C + Bilobalide)% (2)

### 3.10. Extraction and Analysis of Aroma Components

Ginkgo tea (Brand I, 4 g) was put into a heat preservation cup, 200 mL boiling water was added into the same cup and kept warm for 15 min. The tea infusion was filtered immediately into separating funnel and kept closed. After the tea infusion was cooled, aroma components in the tea infusion were extracted using ether. The aroma components were collected for detection after ether was volatilized (40 °C in sand bath pot) using GC/MS. Chromatography column is TR-5MS capillary column, heating program of column temperature box: keep 50 °C for 2 min, heat up to 260 °C by 20 °C/min and keep for 5 min, inlet splitter 250 °C. Carrier gas of 99.999% high purity helium, carrier gas flow rate of 1 mL/min. Mass spectrum conditions: EI ion source, the temperature of the ion source is 250 °C, the ionization voltage is 70 eV, the interface temperature is 250 °C, and the quadrupole temperature is 150 °C.

### 3.11. Enzyme Treatment of Ginkgo Tea

Ginkgo tea (4 g) was put into a heat preservation cup, 200 mL boiling water was added into the same cup and kept warm for 15 min. α-R was added and kept at 65 °C for 1 h in water bath, then β-G was added and kept at 90 °C for 1 h in water bath. The tea infusion was collected by filtration and the glycosidase were precipitated by methanol. The supernatant was prepared as described before for the further analysis.

### 3.12. ABTS Radical Scavenging Activity

The experiment was taken according to the instruction of the assay kit (Beyotime, China) [16]. Briefly, ABTS solution (200 µL) and sample (10 µL) were added to each well of a 96-well plate. The plate was gently mixed and incubated at room temperature for 5 min. The sample absorbance (a), ethanol absorbance (b), and control absorbance (c) were measured at 734 nm. The ABTS^+^ reducing capacity was calculated using the following formula. All experiments were performed with at least four parallels and repeated three times.

Scavenging capacity (%) = (1 − (a − b)/c) × 100% (3)

### 3.13. Free Radical (DPPH) Scavenging Experiment

The DPPH scavenging capacity was measured as previously described with slight modifications [16]. Briefly, the sample (100 µL) was added into DPPH solution (0.5 mM DDPH solution diluted in 95% ethanol, 100 µL) and incubated at room temperature for 30 min in 96-well plate. The sample absorbance (a), ethanol absorbance (b), and control absorbance (c) were measured at 517 nm. The DPPH scavenging capacity was calculated using the above formula.

### 3.14. Lymphocyte Transformation

The experiment was done as mentioned before [16]. Primary lymphocytes (1 × 10^7^/mL, 100 µL) were seeded into 96-well plates. Con A (5 μg/mL, 50 µL) and samples of different concentration (50 µL) were added into each well sequentially. Cells were cultured in the CO_2_ incubator at 37 °C for 48 h. MTT assay was used for the proliferation detection of lymphocytes.

### 3.15. MTT Assay

The experiment was done as mentioned before [41]. MTT solution (4 mg/mL in PBS) was added (20 μL/well) into each well and incubated with cells for 4 h at 37 °C. Then, the supernatant was discarded and the purple formazan crystals were dissolved in 200 μL of DMSO for 5 min. In the end, the plates were read on an automated microplate spectrophotometer (Molecular Devices, San Jose, CA, USA) at 540 nm.

### 3.16. Determination of NO Production by Griess Method

The experiment was done as mentioned before [35]. RAW264.7 cells (2 × 10^5^/mL, 100 μL) were counted and inoculated in a 96-well plate. Twenty-four hours later, the cells were treated with different concentrations of compounds and LPS (500 ng/mL). Three pores were used for each same test and the culture medium containing DMSO was used as a control. After 48 h of incubation, the supernatant (100 μL) was transferred to a new plate and mixed with Griess reagent (100 μL) for 10 min. The absorbance of the mixture was measured at 540 nm. The inhibitory effect of NO release was calculated using the following formula:

NO inhibition rate (%) = (OD_LPS_ − OD_LPS+sample_)/(OD_LPS_ − OD_blank_) × 100% (4)

### 3.17. In Vitro Proliferation Experiment

The experiment was done as mentioned before [22]. Tumor cells were treated with the concentrate of Ginkgo tea infusion for 72 h in 96-well plates. The proliferation of cells was determined by MTT assay.

### 3.18. Statistical Analysis

Data are expressed as mean ± SD. Student’s *t* test was used for statistical analyses of the data. All statistical analyses were conducted using SPSS 10.0 statistical software (SPSS, Chicago, IL). Cases in which *p* values of <0.05 were considered statistically significant [42].

## 4. Conclusions

Although the addition of β-G and α-R brings benefits for making Ginkgo tea, there are still some unsolved problems. For example, different optimum reaction temperatures of the two glycosidases makes it complicated to make Ginkgo tea. Now, we have cloned and expressed another thermostable α-R (the optimum reaction temperature is 90 °C) from *Thermotoga petrophila* DSM13995. However, the expression and purification of this α-R were difficult and the enzyme activity was not ideal. In our future study, we will implement changes at the molecular level to improve the thermal stability, enzyme activity, expression and purification of α-R. In this paper, we only discussed the effect of β-G and α-R on flavonoids, ginkgolides, and aroma components in Ginkgo tea drink. If we want to drink tea directly, a method to separate the enzyme from the tea infusion should be carefully studied (such as Immobilized enzyme technique or Ultrafiltration technique). Taken together, the present study suggests that β-G and α-R have potential application prospects in the making of Ginkgo tea or tea drink.

## Figures and Tables

**Table 1:** Composition and contents of aroma components in the tea infusion with and without β-G/α-R treatment.

Aroma Component	Content (%)			
	Control	β-G	α-R	α-R & β-G
3-Methylbutyric acid	/	/	0.51 ± 0.26	4.4 ± 1.65
Trimethylsilylmethanol	1.35 ± 0.99	/	0.44 ± 0.35	/
2-Methoxy-1,3-dioxolane	2.02 ± 1.52	/	7.84 ± 1.99	8.54 ± 2.58
Dihydroactinidiolide	4.49 ± 1.46	0.795 ± 0.92	0.63 ± 0.28	/
Vanillic acid	1.66 ± 1.19	2.07 ± 1.33	1.16 ± 0.80	4.02 ± 1.14
Vanillin	/	0.61 ± 0.47	2.28 ± 0.89	1.45 ± 0.81
Benzyl alcohol	/	2.07 ± 1.55	/	4.94 ± 2.33
p-Hydroxybenzyl alcohol	/	6.08 ± 1.69	/	1.04 ± 1.00
p-Hydroxybenzaldehyde	/	4.89 ± 2.85	0.29 ± 0.24	8.78 ± 1.83
Dihydro-β-ionone	/	3.77 ± 1.89	/	/
3,4,5-Trimethoxybenzyl alcohol	/	0.54 ± 0.54	/	/
5-Hydroxymethylfurfural	/	0.59 ± 0.28	0.87 ± 0.27	0.72 ± 0.26
Phenylacetic acid	/	/	0.25 ± 0.12	/
p-Hydroxyphenylethanol	/	0.645 ± 0.31	/	0.8 ± 0.58
Piperonylacetone	/	0.42 ± 0.32	2.29 ± 1.18	/
Total	9.52 ± 2.24	22.47 ± 3.96	16.56 ± 4.36	34.69 ± 5.21

Data are mean ± SD, *n* = 3.

**Table 2:** Changes in the contents of characteristic flavone aglycones by enzyme treatment during the making of the tea.

	Aglycone	Concentration (μg/mL) of Flavonoids in the Concentrated Tea Infusion					
		First Time		Second Time		Third Time	
		Control	Enzyme	Control	Enzyme	Control	Enzyme
Brand I	Quercetin	3.15 ± 1.95	149.68 ± 20.15	1.82 ± 0.76	142.49 ± 18.64	2.25 ± 1.43	49.88 ± 11.77
	Kaempferol	1.54 ± 0.66	106.41 ± 13.52	2.10 ± 0.92	105.38 ± 12.01	3.38 ± 1.53	91.93 ± 10.14
	Isorhamnetin	1.17 ± 0.45	97.47 ± 11.04	1.31 ± 0.55	96.77 ± 11.24	1.27 ± 0.52	29.79 ± 4.86
Brand II	Quercetin	3.05 ± 1.34	143.97 ± 16.15	1.94 ± 0.89	107.58 ± 11.56	1.89 ± 0.61	73.16 ± 8.37
	Kaempferol	3.71 ± 1.73	167.12 ± 21.61	2.97 ± 1.30	134.02 ± 14.26	1.75 ± 0.59	142.70 ± 14.77
	Isorhamnetin	2.40 ± 0.98	167.14 ± 20.17	2.02 ± 0.91	125.62 ± 12.94	2.37 ± 0.87	103.34 ± 11.21
Brand III	Quercetin	4.09 ± 2.23	238.10 ± 26.42	2.59 ± 1.16	22.33 ± 4.01	0.44 ± 0.16	8.53 ± 2.79
	Kaempferol	3.78 ± 1.82	170.31 ± 17.52	2.93 ± 1.26	43.94 ± 5.24	3.95 ± 1.86	49.50 ± 6.11
	Isorhamnetin	1.73 ± 0.69	106.19 ± 11.38	1.09 ± 0.44	9.75 ± 2.85	0.79 ± 0.21	5.53 ± 2.35
Brand IV	Quercetin	0.95 ± 0.26	157.06 ± 16.85	0.87 ± 0.32	53.55 ± 6.13	1.32 ± 0.53	8.73 ± 3.04
	Kaempferol	3.10 ± 1.53	187.48 ± 21.33	1.16 ± 0.43	88.84 ± 8.24	2.89 ± 1.03	76.97 ± 7.21
	Isorhamnetin	1.81 ± 0.87	145.34 ± 15.04	0.31 ± 0.14	59.43 ± 6.44	1.42 ± 0.56	14.77 ± 3.56

Data are mean ± SD, *n* = 3.

**Table 3:** Information of each glycosidase.

	TpeBgl3 [24]	TthBgl3 [23]	AniBgl3 [37]	AteRha78 [25]	AniRha78 [38]	BthRha [39]
Type	GH3	GH3	GH3	GH78	GH78	GH78
Optimum temperature (°C)	90	95	60	65	35	55
Optimum pH	5.0	5.0	4.0	6.5	6.5	6.5
Km (mM)	1.6	0.065	0.643	0.476	4.23	2.87
Kcat (s^−1^)	/	121	/	412	/	1743
Vmax (μmol/min·mg)	109	/	0.71	/	3.64 × 10^−3^	/
Substrate specificity	PNPG ^1^	PNPG	PNPG	PNPR ^2^	PNPR	PNPR
Heat stability (h) ^2^	3	1	0.5	3	2	1

^1^ PNPG: *p*-Nitrophenyl-β-d-glucopyranoside; ^2^ PNPR: *p*-Nitrophenyl-α-l-rhamnopyranoside; ^3^ Heat stability means the hours that: the activity of recombinant enzyme TpeBgl3 remained more than 50% at 90 °C; The half-life of the recombinant enzyme TthBgl3 at 90 °C; the activity of recombinant enzyme AniBgl3 remained more than 85% at 60 °C; the activity of recombinant enzyme AteRha78 remained more than 50% at 65 °C; the activity of the recombinant enzyme AniRha78 remained more than 80% at 25, 30 and 35 °C; the half-life of recombinant enzyme BthRha at 50 °C.

## Supplementary Materials

The following are available online, Table S1: The conversion rate of rutin by different combinations of β-glucosidase and α-rhamnosidase. Figure S1: The ginkgolide A, B, C and bilobalide can be released into the Ginkgo tea infusion with repeated brewing. Figure S2: The conversion rate of rutin and yield of quercetin by β-G (TpeBgl3) and α-R (AteRha78) under different catalytic conditions. Figure S3: The amount of ginkgolide A, B, C and bilobalide released into the tea infusion by addition of β-G (0.125 U/mL) and α-R (0.45 U/mL) during making tea. Figure S4: Effect of enzyme dosage on the composition of characteristic flavonoids in the tea infusion. Figure S5: No cytotoxicity of the Ginkgo tea was observed in the normal T cell.
