# Supplementary material for: Effects of β-glucosidase and α-rhamnosidase on the Contents of Flavonoids, Ginkgolides, and Aroma Components in Ginkgo Tea Drink
Source: Molecules. 2019 May 25;24(10):2009. doi: 10.3390/molecules24102009 (PMC6572456; doi:10.3390/molecules24102009)
Supplement: Supplementary file 1 [file molecules-24-02009-s001.pdf]

Supplementary materials

Effects of β-glucosidase and α-rhamnosidase on the contents of flavonoids, ginkgolides and aroma components in Ginkgo tea drink

Xianying Fang [1], Yurong Dong [1], Yingying Xie [1], Lei Wang [1], Jingqiu Wang [1], Yuechen Liu [2], Linguo Zhao [1,3,*] , Fuliang Cao [3]

METHODS

Determine the best combination of β-glucosidase (β-G**) and α-rhamnosidase (**α-R**).** Lutin (4 mg) was dissolved in 200 ml hot water (70℃), then mixed with β-G (45 U) and α-R (12.5 U) and kept in 70 ℃ water bath for 60 min. The reaction was terminated by adding 100 ml methanol. The supernatant was harvested, then concentrated by rotary evaporator and dried by vacuum freeze dryer. Samples were dissoveled in methanol, the content of rutin was determined by HPLC analysis and calculated according to the standard curve. The conversion rate of rutin was calculated by the following formula:

$$\text{Conversion rate (\%)} = (4 - \text{lutin content in sample}) / 4 \times 100\%$$

The condition optimization on enzyme dosage and reaction time for the conversion of rutin by β-G (TpeBgl3) and α-R (AteRha78). Each copy of rutin (10 mg) were dissolved in 200 ml hot water and kept at 65℃ in shaking bath. Firstly, rutin were treated with α-R (5, 25 and 100 U, respectively) and kept at 65℃ for 30 min or 1 h. Then, the mixture were treated with β-G (18, 90 and 360 U, respectively) and kept at 90℃ for 30 min or 1 h. If rutin was treated with α-R for 30 min (or 1 h), the mixture was treated with β-G for 30 min (or 1 h). The content of rutin and quercetin were determined by HPLC analysis. The theoretical 100% yield of quercetin was calculated according to the molar number of rutin.

Cytotoxicity assay. Lymphocyte cells (5×10^5/well) were treated with Ginkgo tea sample of different concentration for 24 h in 96-well plates. The survival of lymphocytes was determined by MTT assay.

Table S1. The conversion rate of rutin by different combinations of β-glucosidase and α-rhamnosidase.

	TpeBgl3	TthBgl3	AniBgl3
AteRha78	95%	90%	22%
AniRha78	11%	9%	6%
BthRha	7%	5%	4%

FIGURES

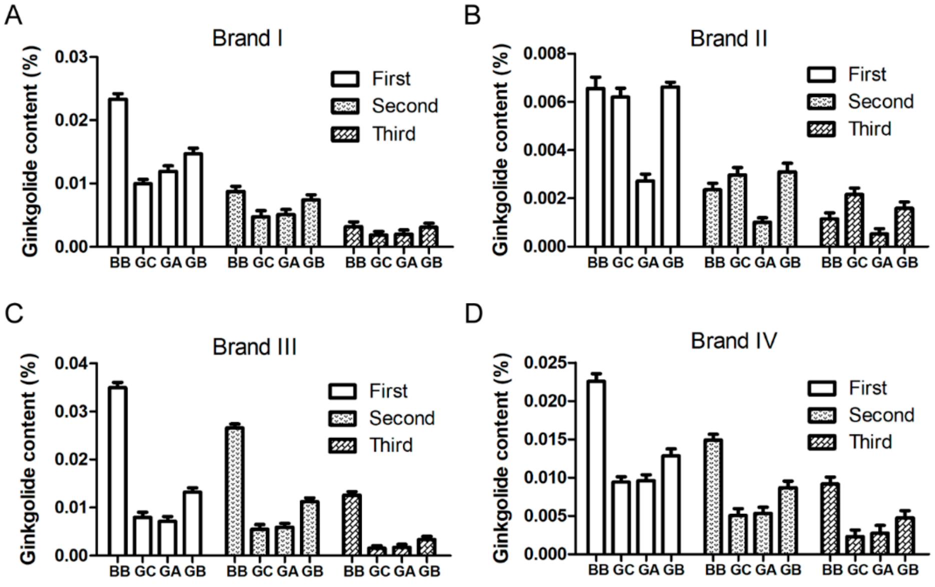

Figure S1. The ginkgolide A, B, C and bilobalide can be released into the Ginkgo tea with repeated brewing. The ginkgolide A, B, C and bilobalid contents in the tea water of Brand I (A), II (B), III (C), and IV (D) were determined by HPLC-ELSD analysis.

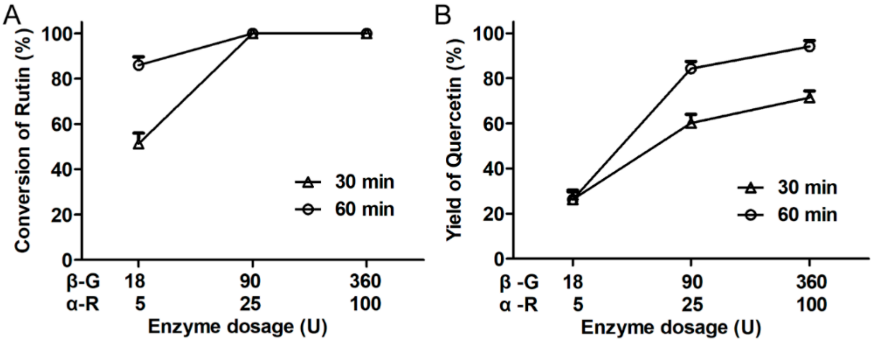

Figure S2. The conversion rate of rutin and yield of quercetin by β-G (TpeBgl3) and *α*-R (AteRha78) under different catalytic conditions. Each copy of rutin (10 mg) were dissolved in 200 ml hot water and kept at 65℃ in shaking bath. Firstly, rutin were treated with *α*-R (5, 25 and 100 U, respectively) and kept at 65℃ for 30 min or 1 h. Then, the mixture were treated with β-G (18, 90 and 360 U, respectively) and kept at 90℃ for 30 min or 1 h. The content of rutin and quercetin were determined

by HPLC analysis and calculated according to the standard curve. (A) The conversion rate of rutin under different enzyme dosage and reaction time. (B) The yield of quercetin under different enzyme dosage and reaction time. Data are mean ± SD of three independent experiments.

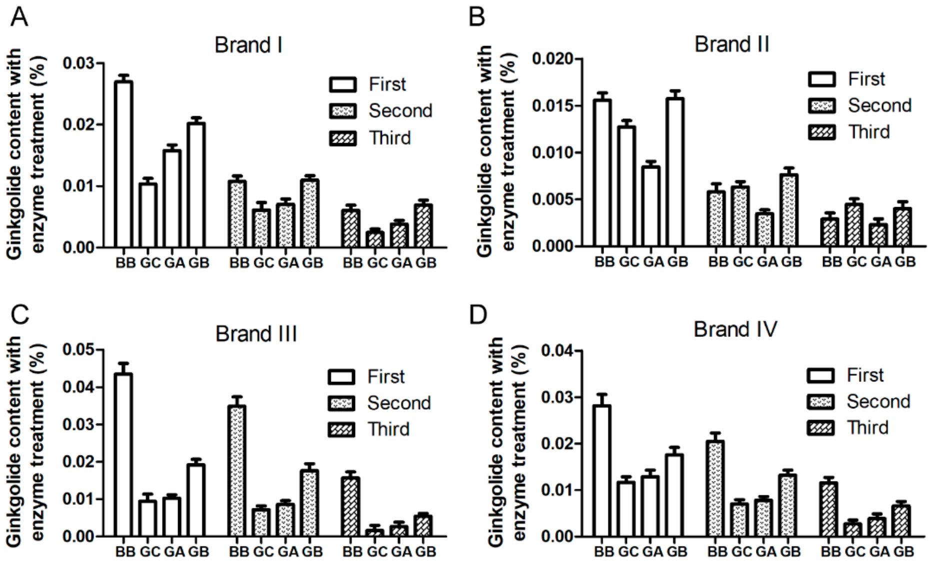

Figure S3. The amount of ginkgolide A, B, C and bilobalide released into the tea infusion by addition of β-G (0.125 U/ml) and α-R (0.45 U/ml) during making tea. Ginkgo tea (4 g) was put into a heat preservation cup, 200 ml boiling water was added into the same cup and kept warm for 15 min. α-R was added and kept at 65℃ for 1 h, then β-G was added and kept at 90℃ for 1 h. Tea infusion of the first time was collected by filtration. 200 ml boiling water was added into the residue of Ginkgo tea and kept warm for 15 min for the second time. Tea infusion of second time was collected by filtration. The above step was repeated for the third time, and the tea infusion of third time was collected by filtration. The ginkgolide A, B, C and bilobalid contents in the tea water of Brand I (A), II (B), III (C), and IV (D) were determined by HPLC-ELSD analysis.

A

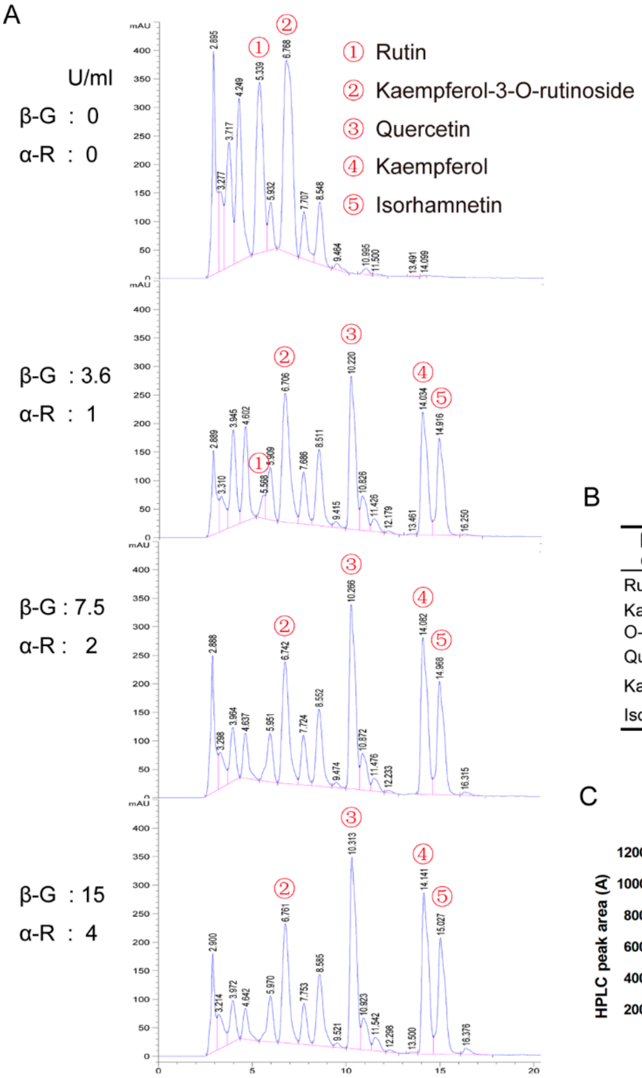

B

β-G (U/ml)<br>α-R (U/ml)	0<br>0	3.6<br>1	7.5<br>2	15<br>4
Rutin	7797.11	667.56	0.00	0.00
Kaempferol-3-O-rutinoside	11088.40	6309.39	5423.30	5094.00
Quercetin	0.00	5960.81	7138.43	7533.24
Kaempferol	14.29	5181.68	6562.79	6787.88
Isorhamnetin	0.00	4446.45	5178.73	5429.64

C

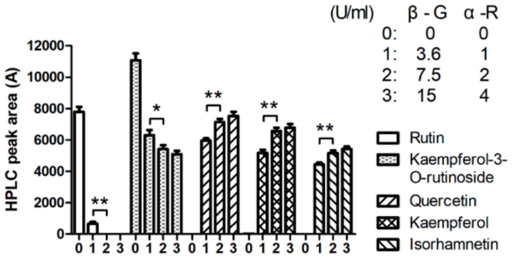

Figure S4. Effect of enzyme dosage on the composition of characteristic flavonoids in the tea. Ginkgo tea (4 g) was put into a heat preservation cup, 200 ml boiling water was added into the same cup and kept warm for 15 min. *α*-R (0, 1, 2 or 4 U/ml) was added and kept at 65℃ for 1 h, then β-G (0, 3.6, 7.5 or 15 U/ml) was added and kept at 90℃ for 1 h. Tea infusion was collected by filtration. Before HPLC detection, the tea infusion was concentrated and dried, then dissolved in methanol. (A) Composition analysis of flavonoids in the tea water by HPLC. (B-C) The peak area of two flavone glycosides and three flavonoid aglycones in the tea. Data are mean ± SD of three independent experiments. **p*<0.05, ***p*<0.01.

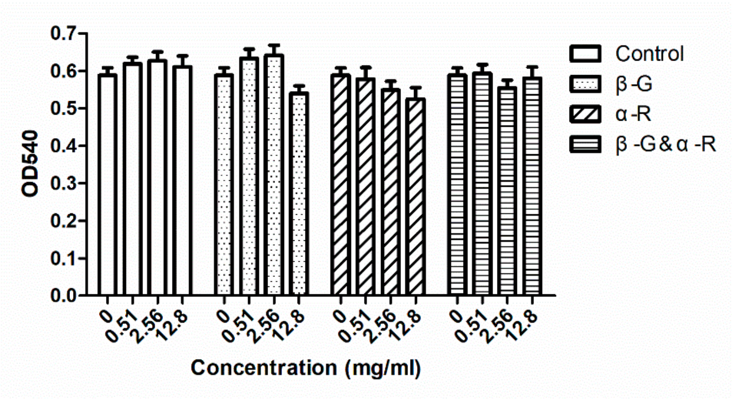

Figure S5. No cytotoxicity of the Ginkgo tea was observed in the normal T cell. Ginkgo tea (4 g) was put into a heat preservation cup, 200 ml boiling water was added into the same cup and kept warm for 15 min. α-R (2 U/ml) was added and kept at 65℃ for 1 h, then β-G (7.5 U/ml) was added and kept at 90℃ for 1 h. The Ginkgo tea was brewed for three times as mentioned before. The experiments in control group were taken with the same steps except that without enzyme addition. The experiments in single enzyme groups were taken with the same steps except that only one kind of enzyme was added. Tea infusion was collected by filtration, then concentrated and dried, dissolved in DMSO. The concentrates were applied for the proliferation assay of naive T lymphocytes by MTT method. The concentration unit 'mg/ml' means that x mg dry raw material (Ginkgo tea) per milliliter. Data are mean ± SD of three independent experiments.
